# The First Reported Case of Elizabethkingia anophelis From Nepal

**DOI:** 10.7759/cureus.45346

**Published:** 2023-09-16

**Authors:** Sharmila Chaudhary, Ashes Rijal, Piyush Rajbhandari, Achyut Bhakta Acharya

**Affiliations:** 1 Critical Care Medicine, Patan Academy of Health Sciences, Kathmandu, NPL; 2 Anesthesiology and Critical Care, Institute of Medicine, Tribhuvan University Teaching Hospital, Kathmandu, NPL; 3 Public Health Sciences, Nepal Health Frontiers, Kathmandu, NPL; 4 Microbiology, Patan Academy of Health Sciences, Kathmandu, NPL; 5 Pulmonary, Critical Care & Sleep Medicine, B.P. Koirala Institute of Health Sciences, Dharan, NPL

**Keywords:** infectious disease medicine, first case from nepal, matrix-assisted laser desorption ionization-time of flight (maldi-tof), critical care and internal medicine, elizabethkingia anophelis infection

## Abstract

*Elizabethkingia anophelis*, a gram-negative bacillus belonging to the Flavobacteriaceae family, is found in various environmental sources and has been associated with community and hospital outbreaks. Correct identification is crucial, guided by advanced genomic techniques, i.e., matrix-assisted laser desorption ionization-time of flight mass spectrometry (MALDI-TOF MS) system with an updated database. The case fatality rate, ranging from 24 to 60%, underscores the need for timely recognition and appropriate management. Additionally, *Elizabethkingia* presents a challenge due to its recent discovery, misidentification history, and drug resistance. Here, we present a case of fatal infection in a 30-year-old male, who presented with pneumonia. It gradually progressed and ultimately proved fatal underscoring the virulence of the pathogen involved. It was a diagnostic challenge as it likely is the first reported instance of *Elizabethkingia anophelis *infection from Nepal.

## Introduction

*Elizabethkingia anophelis* is a gram-negative, aerobic, pale yellow-pigmented, non-motile, glucose-non-fermenting, non-spore-forming, oxidase-positive, weakly indole-positive, and nitrate-negative bacillus belonging to the genus Elizabethkingia and family Flavobacteriaceae [1]. They are widely found in our environment from sources such as water, soil, and insects. It was first isolated from the midgut of the mosquito *Anopheles gambiae* G3, originating from McCarthy Island, The Gambia, in 2011 [2]. Since the first reported human infection was identified in the Central African Republic in a neonate in 2011 [3], multiple community outbreaks as well as hospital outbreaks have been reported. *Elizabethkingia anophelis*, notorious for antibiotic resistance, has been one of the deadliest micro-organisms with high case fatality rates [4,5]. In this case report, we present the case of a middle-aged male who succumbed fatally to the infection, which, to the best of our understanding, remains the first reported case of *Elizabethkingia anophelis* from Nepal. 

## Case presentation

**Figure 1:**
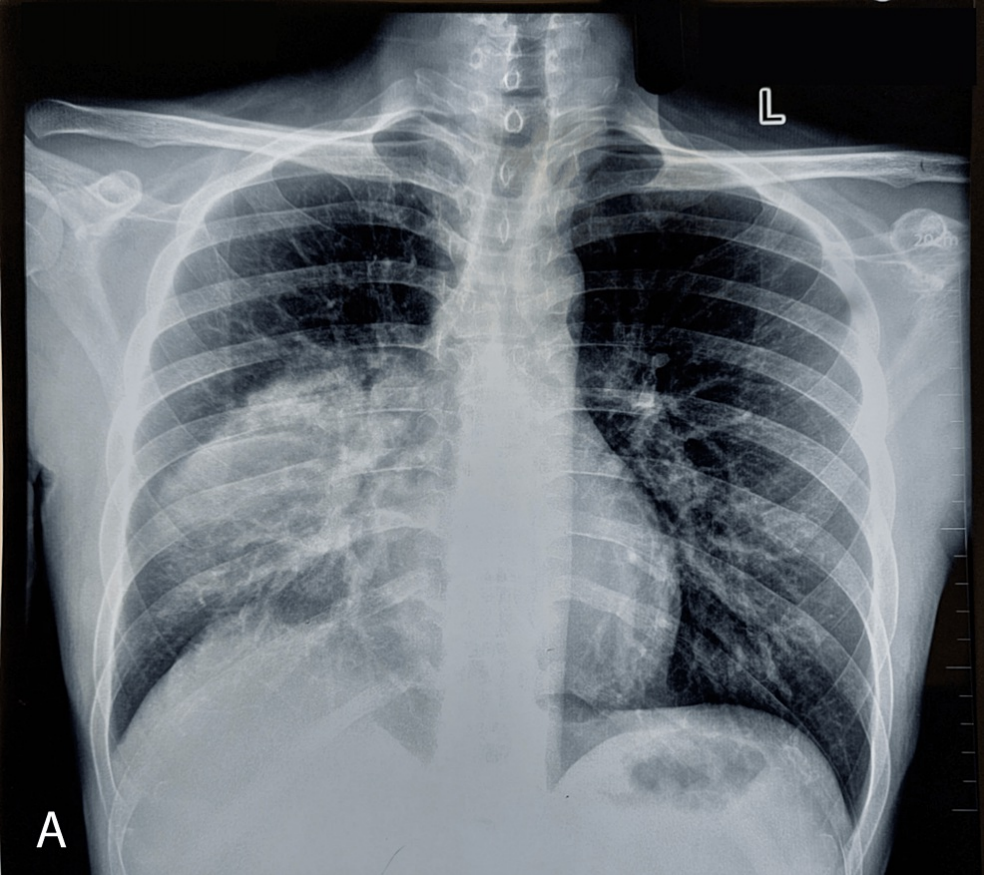
Initial chest X-ray (day 1).

A 30-year-old male from central Nepal initially presented with cough, shortness of breath, and central chest pain for four days. He was a chronic alcohol consumer for five years (34.5 units per week), and his last intake was five days prior to the Patan Academy of Health Sciences (PAHS) emergency visit. He later developed fever, confusion, restlessness, and fluctuating cognition during the hospital stay. On examination, he was not oriented to time, place, or person and was restless and tremulous. He had no signs of chronic liver disease, i.e., spider nevi, Dupuytren's contracture, parotid swellings, gynecomastia, etc. On chest examination, there were decreased breath sounds on the right side. A chest X-ray was done, which showed consolidation on the right middle lobe (Figure 1). A psychiatric consultation was done, and he was evaluated and diagnosed with alcohol-dependent syndrome (ADS) with delirium due to a general medical condition (mixed etiology). He was admitted to the medical high dependency unit (HDU) with a diagnosis of right-sided pneumonia. Following admission, he continuously developed a fever and had increasing oxygen requirements. On the second day of HDU admission, he developed respiratory distress and required 10 liters of oxygen with a reservoir mask. Thus, he was shifted to the medical intensive care unit (MICU).

**Figure 2:**
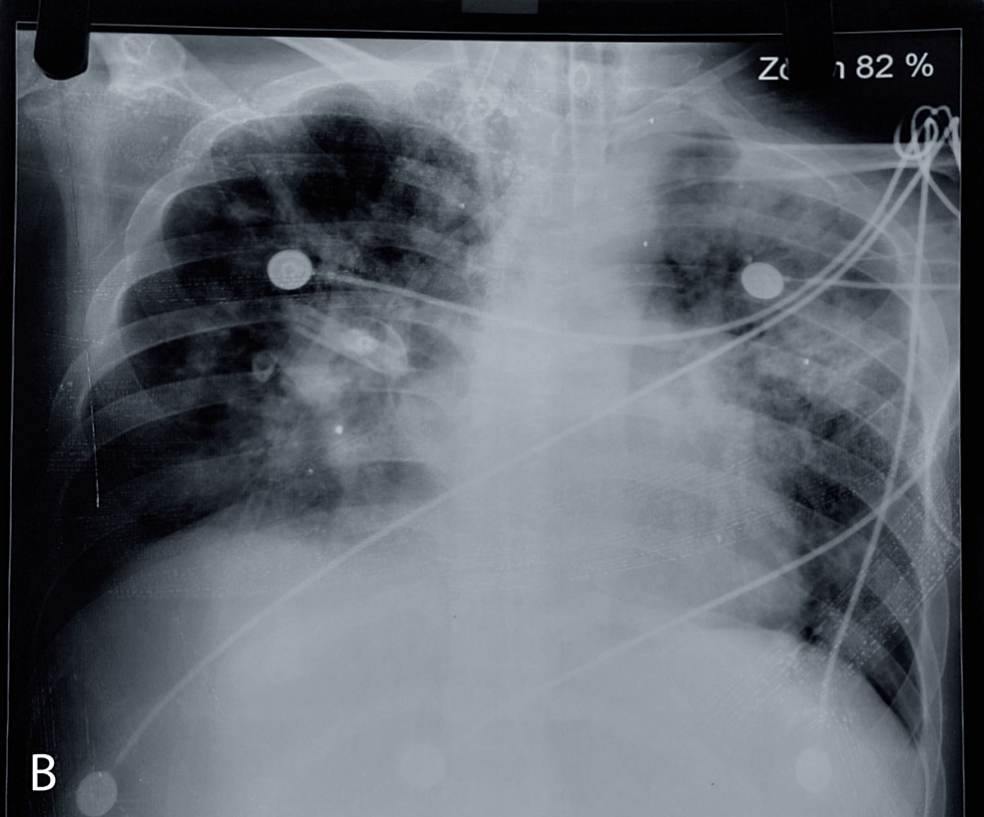
Last X-ray taken (day 28).

Arterial blood gas analysis (ABG) showed metabolic acidosis and type 1 respiratory failure (pH: 7.31, pCO_2_: 28, PO_2_: 58, HCO_3_: 16), and thus he was intubated and mechanically ventilated. He developed pleural effusion, so thoracocentesis was done and sent for analysis. Bacteriological investigations were sent, including endotracheal (ET) aspirate. The isolated organism from ET aspirate was a gram-negative bacillus, but it was growing only on blood agar and not on MacConkey agar. It was deemed difficult to identify with conventional identification methods, and the isolate was sent to the PAHS referral laboratory. The referral laboratory National Public Health Laboratory (NPHL) identified the isolated organism as *Elizabethikingia anophelis* using the matrix-assisted laser desorption ionization-time of flight mass spectrometry (MALDI-TOF MS) system. The organism was subjected to broth microdilution minimum inhibitory concentration (MIC) using VITEK-2, a compact system, which showed the organism was sensitive to ciprofloxacin (MIC 0.5 µg/ml), levofloxacin (MIC 0.5 µg/ml), tigecycline (MIC 2 µg/ml), and minocycline (MIC 1 µg/ml), intermediately sensitive to gentamicin (MIC 8 µg/ml), cefoperazone-sulbactam (MIC 32 µg/ml) and resistant to piperacillin-tazobactam(MIC ≥ 128 µg/ml), ceftazidime (MIC ≥ 64 µg/ml), imipenem (MIC ≥ 16 µg/ml), meropenem (MIC ≥ 16 µg/ml), amikacin (MIC ≥ 64 µg/ml), and colistin (MIC ≥ 16 µg/ml). Blood culture showed growth of coagulase-negative Staphylococcus is sensitive to doxycycline, gentamicin, linezolid, and vancomycin, and urine culture showed growth greater than 10^5^ CFU/ml of yeast cells. The pleural fluid culture showed no growth. Chest X-ray showed further increasing chest consolidation involving the left upper lobes (Figure 2). Bedside echocardiography was done, which showed mild mitral and tricuspid regurgitation and an estimated ejection fraction of 23% with no vegetation. Ultrasonography of the abdomen and pelvis showed features suggestive of early cirrhotic changes in the liver. On the 11th day of MICU admission, he underwent hemodialysis for acute renal impairment. A total of six dialyzes were done during his stay at MICU. On the 24th day of MICU admission, he underwent a tracheostomy for prolonged intubation. Details of his investigations are mentioned in Table 1. 

**Table 1:** Investigation findings. WBC: white blood cells, µ liters: microliters, mg/dl: milligram per deciliters, ET: endotracheal, AFB: acid-fast bacilli, U/liters: unit per liters, gm/dl: gram per deciliters, ADA: adenosine deaminase, HIV: human immunodeficiency virus, LDH: lactate dehydrogenase.

Specimen	Examination	Day 1	Day 8	Day 11	Day 17	Day 23	Day 28
Blood	Total WBC count	6120 cells/µ liters	13000 cells/µ liters	12000 cells/µ liters	14400 cells/µ liters	15910 cells/µ liters	13110 cells/µ liters
Blood	Hemoglobin	10.4 mg/dl	9.5 mg/dl	8.6 mg/dl	7.4 mg/dl	8.9 mg/dl	7.3 mg/dl
Blood	Urea	30 mg/dl	34 mg/dl	63 mg/dl	194 mg/dl	118 mg/dl	98 mg/dl
Blood	Creatinine	0.5 mg/dl	0.4 mg/dl	2.3 mg/dl	5 mg/dl	2.5 mg/dl	1.7 mg/dl
Sputum	Culture	No pathogen was isolated after 48 hours of incubation.					
ET aspirate	Culture			Growth of gram-negative pathogen but not identified.	*Elizabethkingia anopheles *isolated.		
Sputum	AFB stain	AFB not seen.		AFB not seen.			
Sputum	Gene expert	*M. tuberculosis* not detected.		*M. tuberculosis* not detected.			
Sputum	Gram stain	Normal upper respiratory flora is seen.		Normal upper respiratory flora is seen.			
Blood	Culture			Coagulase-negative *Staphylococcus* isolated.	Coagulase-negative *Staphylococcus* isolated.		
Urine	Culture	No growth was seen after 48 hours of incubation.		No growth was seen after 48 hours of incubation.			
Pleural fluid	Total WBC count		2350 cells/µ liters, lymphocyte: 30%, polymorphs: 70%				
Pleural fluid	LDH		1448 U/liters				
Pleural fluid	Protein		2.4 gm/dl				
Pleural fluid	ADA		25 U/liters				
Pleural fluid	Culture		No growth was seen after 48 hours of incubation.				
Serum	Serology for Hepatitis C, Hepatitis B, HIV	Non-reactive					

The patient was initially treated with an injection of piperacillin-tazobactam 4.5 gm three times a day for eight days. As the medical condition didn't improve and no bacteria was isolated initially, the antibiotics were upgraded to injection imipenem 250 mg twice a day, tablet cotrimoxazole 960 mg once a day, and injection colistin two million units twice a day. Injection vancomycin 1 gm once a day was added after the isolation of coagulase-negative Staphylococcus. Tablet cotrimoxazole was then stopped. Following isolation of *E. anophelis,* injection levofloxacin 500 mg twice a day was added as per drug sensitivity reports, and injections of colistin and imipenem were stopped after seven days of use. On the 29th day, he succumbed to his illness.

## Discussion

*Elizabethkingia anophelis *is one of the six species under the genus *Elizabethkingia* [1]. It was first isolated in 2011 from the midgut of *Anopheles gambiae* C3, which was soon followed by the first human infection in the same year [2,3]. But looking at it retrospectively, it is thought that many cases of *Elizabethkingia anophelis* were misidentified as *Elizabethkingia meningosepticum* with prior MALDI-TOF MS systems commercial reference databases. The reference database has since been updated. 16s-rRNA genome sequencing remains more reliable in species identification [1,4]. Since 2011, many outbreaks have been reported related to *E. anophelis*. Most notably in 2015-2016 in the United States in the states of Wisconsin (63), Illinois (2), and Michigan (1), of a total of 66 cases. Despite extensive workup, no sources were identified [4]. The researchers have identified the sources of *E. anopheles* from insects, soil, hospital water sources, hand hygiene sink aerators, and medical supply kits (COVID-19 swab kits) [2,6-8]. 

Although first identified in Anopheles mosquitoes, no vector transmission has been noted to date. Nosocomial contact, person-to-person contact, and contamination-based spread seem to be the most common modes of transmission. Interestingly, two cases of vertical transmission of microbes from mother to infant were reported in Hong Kong [9]. It inspires further study of the pathogenesis of the microbe and the formulation of appropriate prevention methods for neonatal infection. Even though most of the cases identified are related to contact with a healthcare setting, new isolated cases in the community without nosocomial contact in immunocompetent individuals have also been identified, further increasing the risk spectrum of infection by *E. anophelis* [10,11]. Our case was a patient with a comorbid condition and admitted to the ICU, which lies in the most common niche of *E. anophelis* infection. The most common presentation of its infection includes bacteremia, pneumonia, catheter-related bloodstream infection, meningitis, skin and soft-tissue infection, urinary tract infection, and biliary tract infection [1]. The patient we encountered developed pneumonia and subsequently succumbed to it. Atypical presentations, including vascular injury and thrombocytopenia due to sepsis with subsequent central nervous system (CNS) hematoma, have also been reported in infants [12]. Given all the studies in consideration of its relatively recent discovery, further studies to solidify its pathogenesis, epidemiology, and public health concerns need to be further addressed. 

The most troublesome effect of the infection with *Elizabethkingia* species has been its drug resistance pattern. *E. anophelis* is resistant to most beta-lactams, carbapenems, and aminoglycosides, including drugs such as vancomycin, ceftazidime, cefepime, aztreonam, ceftazidime/clavulanic acid, cefepime/clavulanic acid, colistin, and fosfomycin, which represent most drugs used in the last line of empirical treatment in hospital-acquired infections [13]. It further emphasizes the need for early and correct identification of species for timely treatment. The drugs that have been shown to be sensitive have been minocycline (100%), piperacillin-tazobactam (71.8%), levofloxacin (38.5%), ciprofloxacin (30.8%), piperacillin (17.9%), rifampicin (20.5%), and tigecycline (10.3%) [13]. This finding aligns with the sensitivity pattern seen in our case with *Elizabethikingia anophelis,* which was sensitive to ciprofloxacin, levofloxacin, tigecycline, minocycline, and intermediately sensitive to gentamicin. This result supports the priority use of minocycline or piperacillin-tazobactam [14]. One thing to be noted while studying drug sensitivity is the disk diffusion method and Etest are not appropriate for antibiotic sensitivity testing (AST) assays for *E. anophelis* due to the high rate of errors. Studies done in South Korea and the USA using disk diffusion test or agar dilution test compared to sensitivity test done using broth microdilution test in Taiwan and Singapore showed large discrepancies [5,13]. Thus, although disk diffusion is an easier and faster method of testing, the broth microdilution test represents the gold standard for antimicrobial sensitivity testing, as was done in our case [13]. Even in our case, the bacteria were not identified in our laboratory and thus were sent to the referral laboratory of Nepal, i.e., the Nepal Public Health Laboratory, which correctly identified it using MALDI-TOF MS systems with an updated database. Thus, raising the question of how many cases as such were previously unidentified. This remains the first reported case of *E. anophelis* from Nepal.

Our patient died following a month-long hospital stay, as is the case in most patients, as the case fatality rate ranges from 24 to 60% [1]. Apart from the comorbid condition and nosocomial contact, anemia is shown to be an independent risk factor. Additionally, the use of β-lactam/lactamase inhibitor antibiotics was significantly higher in patients who did not survive as most are resistant to it [13]. It asks for greater vigilance from the treating physician to look for microbes such as *E. anophelis*,* *which can be a rare cause of such hospital-acquired infection, to timely identify and treat for better patient outcomes. 

## Conclusions

In conclusion, *Elizabethkingia anophelis* is a relatively newly discovered species whose clinical implications are coming to light as emerging research continues to unfold. The challenge of distinguishing it from *Elizabethkingia meningosepticum* in the past has been overcome with advancements in genomic sequencing. The microbe has been associated with various outbreaks, predominantly in healthcare settings, but isolated cases in the community have also been reported. Its drug resistance pattern is concerning, as it shows resistance to many commonly used antibiotics, emphasizing the need for early and correct identification to guide appropriate treatment. The case fatality rate for *E. anophelis* infections is significant, warranting greater vigilance from healthcare providers to timely identify and treat infections caused by this pathogen for improved patient outcomes. Further studies are needed to better understand its pathogenesis, epidemiology, and public health implications.
